# Anthropogenic Aerosols Modulated 20th‐Century Sahel Rainfall Variability Via Their Impacts on North Atlantic Sea Surface Temperature

**DOI:** 10.1029/2021GL095629

**Published:** 2021-12-28

**Authors:** Shipeng Zhang, Philip Stier, Guy Dagan, Minghuai Wang

**Affiliations:** ^1^ Department of Physics Atmospheric, Oceanic and Planetary Physics University of Oxford Oxford UK; ^2^ Institute of Earth Sciences Hebrew University of Jerusalem Jerusalem Israel; ^3^ Joint International Research Laboratory of Atmospheric and Earth System Sciences and School of Atmospheric Sciences Nanjing University Nanjing China

**Keywords:** aerosols, Sahel rainfall, North Atlantic variability, aerosol‐cloud‐interactions

## Abstract

The Sahel rainfall has a close teleconnection with North Atlantic sea surface temperature (NASST) variability, which has separately been shown to be affected by aerosols. Therefore, changes in regional aerosols emission could potentially drive multidecadal Sahel rainfall variability. Here we combine ensembles of state‐of‐the‐art global climate models (the CESM and CanESM large ensemble simulations and CMIP6 models) with observational data sets to demonstrate that anthropogenic aerosols have significantly impacted 20th‐century detrended Sahel rainfall multidecadal variability through modifying NASST. We show that aerosol‐induced multidecadal variations of downward solar radiative fluxes over the North Atlantic cause NASST variability during the 20th century, altering the ITCZ position and dynamically linking aerosol effects to Sahel rainfall variability. This process chain is caused by aerosol‐induced changes in radiative surface fluxes rather than changes in ocean circulations. CMIP6 models further suggest that aerosol‐cloud interactions modulate the inter‐model uncertainty of simulated NASST and potentially the Sahel rainfall variability.

## Key Points

- A process chain from anthropogenic aerosol emissions to the Sahel rainfall is proposed
- Large ensemble and CMIP6 simulations suggest that this link is mediated by aerosol impacts on North Atlantic SST
- Both aerosol‐cloud interactions and aerosol‐radiation interactions are important in this chain process

## 1 Introduction

The Sahel region observed a large multidecadal rainfall variability during the 20th century, with a severe drought from 1950s to 1980s and subsequent recovery up to present day (Held et al., 2005; Wood et al., 2015). This significant multidecadal regional variability caused substantial impacts on the local ecosystem and population and triggered large scientific interest. Previous studies have proposed several drivers of the drying and wetting trends over different periods and drew various conclusions. Proposed mechanisms include the subtropical drying due to the ongoing global warming (Dai, 2011; Held et al., 2005), wetting resulting from the direct warming of North Africa caused by greenhouse gases (GHGs) (Dong & Sutton, 2015; Richardson et al., 2016) or absorbing aerosols (Samset et al., 2016; Zhang et al., 2021), teleconnections with varying regional sea surface temperature (SST) (Jordan et al., 2018; Palmer, 1986) caused by GHGs and aerosols (Hill et al., 2018; Hirasawa et al., 2020) and internal variability (Held et al., 2005; Martin et al., 2014; Monerie et al., 2017; Qin et al., 2020). However, the conclusions generally remained model dependent (Biasutti, 2013; Giannini & Kaplan, 2019), with significant uncertainties on distinguishing contributions from different drivers to the Sahel rainfall variability (Ackerley et al., 2011; Dong & Sutton, 2015; Held et al., 2005; Hirasawa et al., 2020; Jordan et al., 2018; Monerie et al., 2017).

The mechanisms can be further separated into local (non‐SST mediated) and nonlocal (SST mediated) effects (Biasutti et al., 2008; Dong & Sutton, 2015; Hirasawa et al., 2020; Undorf et al., 2018), depending on whether the processes are involved with changes in oceanic properties. External forcers, such as GHGs and aerosols, can lead to a change in local surface temperature, which then lead to an increase or decrease of precipitation, although the relative contribution of each external forcer to real‐world Sahel rainfall remains uncertain (Dong & Sutton, 2015; Hirasawa et al., 2020). As for the SST‐mediated effects, earlier studies suggest that North Atlantic sea surface temperature (NASST) explicitly affects the Hadley cell strength and the ITCZ position (Cvijanovic & Chiang, 2013; Zhang & Delworth, 2006). When the northern hemisphere gets warmer compared to the southern hemisphere, the ITCZ shifts northward, which can in turn dynamically modify the west African monsoon and Sahel rainfall (Biasutti, 2019; Dixon et al., 2018; Hua et al., 2019; Watanabe & Tatebe, 2019). The Atlantic Multidecadal Variability (AMV) has been observed to switch between a negative and positive phase on a decadal timescale during the past century (Booth et al., 2012; Zhang & Delworth, 2006). Aerosols have been proposed to substantially impact this NASST variability, although the mechanisms (via aerosol‐induced changes in radiative fluxes or ocean circulations) have remained uncertain (Booth et al., 2012; Dagan et al., 2020; Menary et al., 2020). Therefore, it could be expected that aerosols could influence Sahel rainfall multidecadal variability via their effect on NASST variability. Here, we focus on the SST‐mediated changes in Sahel precipitation and examine the role of anthropogenic aerosols. We systematically explore the process chain, from regional anthropogenic emissions of aerosols and their precursors, to changes in North Atlantic surface net radiative fluxes, via NASST variability to a shift of ITCZ, and eventually Sahel rainfall variability, in ensembles of state‐of‐the‐art global climate models (GCMs) as well as in observations.

## 2 Methods

### 2.1 Large Ensemble Simulations

This study uses the Community Earth System Model 1 large ensemble simulations (CESM1‐LE) (Kay et al., 2015), with coupled atmosphere, ocean, land, and sea‐ice components. CAM5 is used as the atmospheric component, with a resolution of approximately 1° latitude/longitude and 30 vertical levels. The all‐forcing experiment includes 40 ensemble members, forced with historical external forcing from 1920 to 2005. For XAER (XGHG) experiments, the 20 ensemble members are forced with the same historical forcing but with industrial aerosol (GHG) emission fixed at 1920 level (Deser et al., 2020). Both aerosol direct and indirect effects are included in these simulations. Details of CESM1‐LE can be also found in Kay et al. (2015).

Following previous studies (Dai et al., 2015; Hua et al., 2019), the internal variability (IV) of individual members can be calculated as

(1) $$IV_{i}=\;ALL_{i}-ALL_{em}$$

where the subscript *i* denotes each member and the subscript *em* denotes the ensemble mean of a specific experiment. ALL indicates the all‐forcing experiment. Similarly, we can also obtain a spread of members with anthropogenic aerosols only and greenhouse gases only, with the data sets from XAER and XGHG experiments, following (Hirasawa et al., 2020),

(2) $$AER_{i}=\left(XAER_{i}-XAER_{em}\right)+\left(ALL_{em}-XAER_{em}\right)\;$$

(3) $$GHG_{i}=\left(XGHG_{i}-XGHG_{em}\right)+\left(ALL_{em}-XGHG_{em}\right)\;$$

We also use the large ensembles from the Canadian Center for Climate Modeling and Analysis Canadian Earth System Model 2 (CCCma CanESM2‐LE) (Kushner et al., 2018). We use the all‐forcing experiment which has 50 members with a resolution of approximately 2° latitude/longitude, starting from 1950 to 2020 (noting that the starting year is different from CESM1‐LE).

### 2.2 CMIP6

We also use data sets from the Coupled Model Intercomparision Project Phase 6 (CMIP6). Nine models are included in this work (Table S1 in Supporting Information S1). All participating models are coupled with sea‐ice, ocean, land as well as atmosphere. Specifically, we use simulations from the Detection and Attribution Model Intercomparison Project (DAMIP) (Gillett et al., 2016; http://damip.lbl.gov/experiments). For historical experiments, models simulate the historical period from 1850 to 2020 with all historical forcing. To separate out the effect from a single forcing agent, we further use hist‐aer, hist‐ghg, and hist‐nat experiments. Individually, hist‐aer experiments are subject to only historical anthropogenic aerosol forcing, hist‐ghg experiments subject to only historical greenhouse gases forcing, and hist‐nat subject to only historical natural forcing. We note that the starting year is different from the simulations from CESM1‐LE (year 1920). The emission data set of anthropogenic sulfur dioxide (Hoesly et al., 2018) is from the input4MIPS (input data sets for Model Intercomparison Projects).

### 2.3 Observational Data Sets

Two observational data sets of SST are used in this study, the Hadley Center HadISST (Rayner, 2003) and the NOAA reconstructed SST (Huang et al., 2017) data sets. The HadISST data set is available from 1870 with a 1° resolution. The NOAA SST is available from 1854 with resolution at 2°. We also use the CRU station‐based precipitation observational data set from the Climate Research Unit (Harris et al., 2020) (CRU), University of East Anglia, which covers almost all 20th‐century precipitation measurements over land, at around 2.5° resolution.

## 3 Results

**Figure 1:**
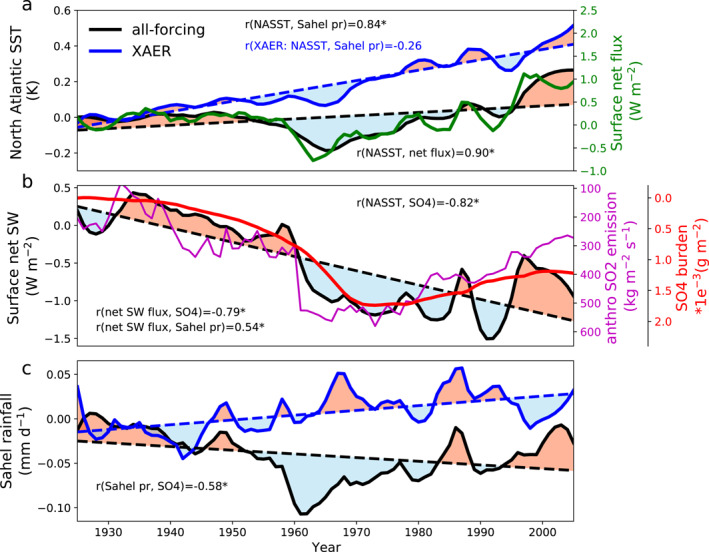
(a) Community Earth System Model 1 large ensemble (CESM1‐LE) simulated North Atlantic (7.5°–75°W, 10°–60°N, see magenta box in Figure 3) sea surface temperature (SST) for all‐forcing simulations (black line) and XAER simulations (blue line). Also shown is the North Atlantic net surface energy flux (defined as downward positive, green line). (b) CESM1‐LE all‐forcing experiments simulated net surface solar radiative flux (black line), and the sulfate aerosol burden (red line, notice the reversed *y* axis) over the North Atlantic Ocean. The purple line indicates the 20th‐century anthropogenic sulfur dioxide emissions from the North Atlantic Ocean as well as East North America and West Europe (95°W–20°E, 10°–60°N, see Figures S2 and S3 in Supporting Information S1 for the regional information). (c) CESM1‐LE simulated ensemble‐mean Sahel (20°W–40°E, 10°–20°N; see black box in Figure 3) rainfall for all‐forcing (black line), and XAER (blue line) experiments. All data sets are shown as the anomalies (10 years low‐pass filtered) relative to the 1920–1925 average. The mark “*” after the correlation coefficient indicates the correlation is significant (*p* value < 0.01). Dashed lines indicate the linear trend and red/blue patches indicate the positive/negative phase of detrended data sets.

**Figure 2:**
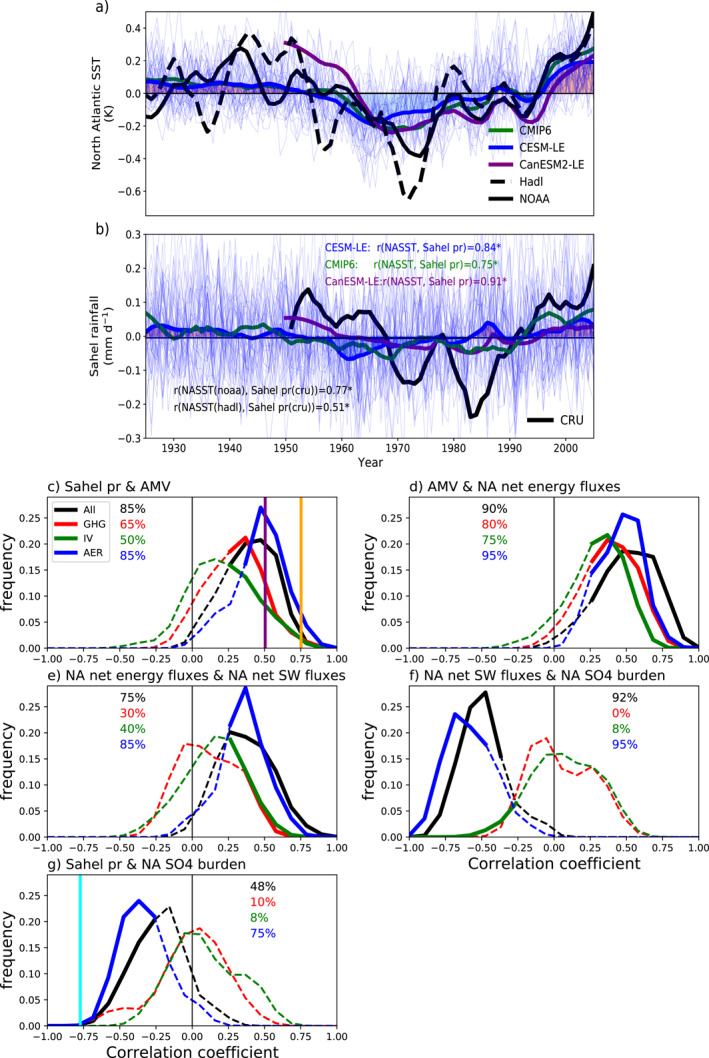
(a) Time series of detrended North Atlantic sea surface temperature (10 years low‐pass filtered), derived from Community Earth System Model 1 large ensemble (CESM1‐LE, blue line), Canadian Earth System Model 2 (CanESM2‐LE, purple line), and Coupled Model Intercomparision Project Phase 6 (CMIP6, green line) ensemble mean as well as observed sea surface temperature (SST) data from Hadley Center HadISST (black dash line) and the NOAA reconstructed SST (black line). Thin blue lines are the individual members from CESM1‐LE. (b) Same as (a) but for detrended Sahel rainfall anomalies. Also shown are observed Sahel rainfall from Climate Research Unit (CRU, black line). All data sets in (a) and (b) are shown as anomalies relative to the 1920–1925 average. The mark “*” after the correlation coefficient indicates the correlation is significant (*p* value < 0.01). The red/blue shading indicates the positive/negative phase of detrended North Atlantic sea surface temperature (NASST) or Sahel rainfall. (c) The estimated possibility density distribution of the correlation coefficients between the CESM1‐LE simulated 1925–2005 (10 years low‐pass filtered) detrended annual Sahel rainfall and NASST from individual ensemble members with all‐forcing (black line), greenhouse only (red line), anthropogenic aerosols only (blue line), and internal variability (green line). Solid lines indicate where the correlations are significant (*p* value < 0.05), while dash lines indicate otherwise. Numbers in percent indicate the fraction of members showing significant correlations. The vertical purple (orange) line in (c) indicates the correlation coefficient between CRU Sahel rainfall and Hadley SST (NOAA SST) from year 1950 to 2005. (d) Same as (c) but for correlations between Atlantic Multidecadal Variability (AMV) and North Atlantic surface net energetic fluxes. (e) Same as (c) but for correlations between North Atlantic surface net energetic fluxes and net shortwave fluxes. (f) Same as (c) but for correlations between North Atlantic surface net shortwave fluxes and SO_4_ burden. (g) Same as (c) but for correlations between Sahel rainfall and North Atlantic SO_4_ burden. The light blue line in (g) indicates the correlation coefficient between CRU Sahel rainfall and CESM1‐LE simulated SO_4_ burden over the North Atlantic Ocean from year 1950 to 2005.

We first examine the ensemble‐mean NASST (10‐year low‐pass filtered) in simulations using transient historical forcings (all‐forcing) in CESM1‐LE. NASST slightly increase during the 20th century (around 0.1 K per century) (Figure 1a), mainly driven by GHG‐induced global warming (Figure S1 in Supporting Information S1) and partly masked by cooling effects from aerosols (Figure 1a). We detrended this long‐term trend to isolate AMV and examine the multidecadal variability on top of the time evolution. The detrended ensemble‐mean NASST shows a pattern of multidecadal variability, with a generally positive phase lasting from 1925 to 1955 and a subsequent negative phase during 1955 and 1985, followed by another positive phase to 2005 (Figure 1a). This pattern exists in observed detrended NASST as well (Figure 2a) with larger magnitude: NASSTs from two data sets indicate that the AMV has gone through a positive‐negative‐positive phase pattern with almost the same timing as suggested by CESM1‐LE results. Although NASST can also be significantly impacted by internal oceanic variability (Knight et al., 2006), this consistency between observations and CESM1‐LE ensemble‐mean (which is expected to eliminate internal variability) results suggests that detrended 20th‐century NASST multidecadal variability emerges due to external forcings. This pattern is consistent with previous work based on a different model (Booth et al., 2012). Furthermore, this pattern does not emerge in ensemble‐mean results averaged over simulations with anthropogenic aerosol emissions fixed at year 1920 levels (XAER) (Figure 1a), which suggests that anthropogenic aerosols are the main driver of the variability in CESM1‐LE.

The strong correlation between NASST and the net surface energy flux (the sum of surface net longwave flux, shortwave flux, sensible, and latent heat flux) over the North Atlantic Ocean (correlation coefficient *r* = 0.90, *p* < 0.01) suggests that the variability of NASST is modulated by the local surface energy budget. Emissions of anthropogenic sulfate aerosols and their precursors from Europe and North America (see Figure S2 in Supporting Information S1 for regional emission) have increased until the 1970s and decreased afterward (purple line in Figure 1b, Smith et al., 2011). Sulfate aerosols reduce downwelling solar radiation and subsequently sea surface temperatures by directly scattering sunlight back to space or indirectly, via interactions with clouds (Bellouin et al., 2020; Boucher et al., 2013; Wild, 2009). These aerosol radiative effects explain the strong relationship between the sulfate burden and the surface net solar radiative fluxes over the North Atlantic Ocean (*r* = −0.79, *p* < 0.01; Figure 1b), which in turn impacts NASST multidecadal variability (correlation coefficient between NASST and sulfate burden *r* = −0.82, *p* < 0.01). Previous studies (Dagan et al., 2020; Menary et al., 2020) also noted that aerosol‐induced changes in oceanic circulations may contribute to NASST variability as well, which we will explore later.

CESM1‐LE ensemble‐mean 20th‐century detrended Sahel rainfall exhibits a multidecadal variability (Figure 1c), consistently in phase with the detrended NASST (*r* = 0.84, *p* < 0.01) as well as the North Atlantic surface solar radiation (*r* = 0.54, *p* < 0.01; Figure 1b). This consistency suggests a relationship between North Atlantic aerosol burden and Sahel rainfall (*r* = −0.58, *p* < 0.01; Figure 1c). A similar pattern is also found in observed detrended 20th‐century Sahel rainfall (Figure 2b), except for a discrepancy from 1950 to 1970, but this could be obscured by internal variability, which is another essential driver of Sahel rainfall variability (Held et al., 2005; Monerie et al., 2017), or due to the fact that early rainfall observation data (before 1950) are sparser and less reliable (Dai et al., 2004). Again, this multidecadal phase‐to‐phase change of Sahel rainfall is not present in XAER simulations, with a much weaker correlation between the Sahel rainfall and NASST (*r* = −0.26, *p* > 0.01). It suggests that the simulated and observed Sahel rainfall multidecadal variability is primarily caused by aerosols. Similar relationships are also evident in CanESM2‐LE (Figure S4 in Supporting Information S1).

Although the increasing long‐term trend of NASST differs from the decreasing long‐term trend of Sahel rainfall, there exists a positive‐negative‐positive pattern of multidecadal variability in both detrended NASST and Sahel rainfall, as suggested by CESM1‐LE all‐forcing ensemble‐mean results (Figure 1). This pattern is also evidenced by CanESM2‐LE (*r* = 0.91), observational data sets (*r* = 0.77 and 0.51, respectively) and CMIP6 (the Coupled Model Intercomparison Project Phase 6) ensemble‐mean results (*r* = 0.75; Figure 2). It is worth noting that although the observational detrended NASST and Sahel rainfall fall into the range estimated by CESM1‐LE members, the ensemble‐mean results largely underestimate the magnitude, which has also been recognized by previous studies (Hirasawa et al., 2020; Hua et al., 2019; Undorf et al., 2018). Nevertheless, the consistent relationship between Sahel rainfall and NASST found in models and observations provides independent support of our conclusions.

Figures 2c–2g give a more quantitative understanding of the process chain, and the role of its different drivers (see Section 2). Most (85% of them) CESM1‐LE all‐forcing ensemble members present a significant positive correlation (*p* < 0.05) between the Sahel rainfall and AMV, as found in observations (orange and purple lines in Figure 2c) and previous studies (Martin et al., 2014; Zhang & Delworth, 2006). AMV is then found to be significantly correlated with the net surface energy flux over the North Atlantic Ocean in the all‐forcing experiments (Figure 2d). The positive correlations arise due to the strong role of NASST in setting the location of the ITCZ (Hua et al., 2019), and this is found for all drivers. Among three drivers, AER, however, shows the most cases with significant correlation, which suggested that aerosols contribute most to the interdecadal variation in AMV and therefore the Sahel rainfall. Furthermore, the variations of net surface energy flux are driven by the net surface shortwave flux variations over the North Atlantic Ocean (Figure 2e), which is further driven by the sulfate burden (Figure 2f). This is only happening for members including the effect of aerosols. Finally, almost half of all‐forcing ensemble members show significant negative correlations between SO= burden over the North Atlantic Ocean and the Sahel rainfall. More than half of the members (75%) with only anthropogenic aerosols also indicate such a significant negative correlation, while other drivers (GHG and IV) do not (Figure 2g). It is interesting to note that the observed CRU Sahel rainfall indicates an even stronger (more negative) correlation (light blue line in Figure 2g—at the edge of the CESM1‐LE distribution). This result suggests the important role of anthropogenic aerosols in the process chain.

**Figure 3:**
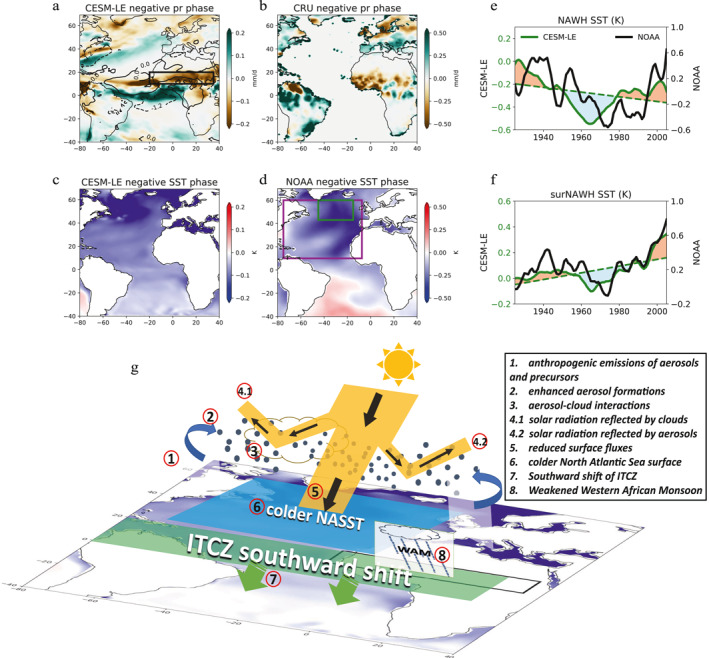
Spatial pattern of grid‐point detrended rainfall averaged over the negative phase (year 1970–1980) period, derived from the (a) Community Earth System Model 1 large ensemble (CESM1‐LE) all‐forcing experiment and the (b) Climate Research Unit (CRU) data set. The black box in (a) indicates the Sahel region. Spatial patterns of grid‐point detrended sea surface temperature (SST) averaging over the negative phase period, derived from the (c) CESM1‐LE all‐forcing experiment and (d) NOAA SST data set. Also shown in (a) is the detrended outgoing longwave radiation (OLR; contour lines) over the same period. The magenta box and the green box in 3 days indicate the North Atlantic region and the North Atlantic warming hole region (NAWH, 15°–45°W, 43°–60°N), respectively. (e) Twentieth‐century timeseries of SST over the NAWH derived from the CESM1‐LE all‐forcing experiment (green line) and the NOAA SST data set (black line; notice the different scale). (f) Twentieth‐century timeseries of SST over the rest of North Atlantic region surrounding NAWHSST (surNAWH) derived from the CESM1‐LE all‐forcing experiment (green line) and the NOAA SST data set (black line). Dashed lines indicate the linear trend, and red/blue patches indicate the positive/negative phase of detrended SST. (g) A schematic plot based on Figure 3c to illustrate the process chain of anthropogenic aerosols effects on Sahel rainfall variability.

We next investigate the mechanisms involved in the process chain from aerosols to Sahel precipitation, showing that this teleconnection acts via the interaction of the ITCZ and the West African monsoon. We sample the CESM1‐LE simulated detrended Sahel rainfall (Figure 3a) and SST (Figure 3c) at a negative phase (year 1970–1980) for both NASST and Sahel rainfall, respectively. It shows that when there is a negative phase of AMV simulated in CESM1‐LE (Figure 3c), the shift of the thermal equator associated with a colder Northern Hemisphere leads to a southward shift of ITCZ (Ridley et al., 2015; Wang, 2009) as well as an accompanied shift of tropical rain belt (Figure 3a), weakening the strength of the West African monsoon, resulting in a negative phase of detrended Sahel rainfall (Figure 3a), and vice versa for the positive phase of AMV (year 1995–2005, see Figure S5 in Supporting Information S1). This teleconnection is also found in observational data sets (Figures 3b and 3d).

Previous studies have already acknowledged the aerosol effects on NASST, but the attribution (from aerosol‐induced changes in radiation or ocean dynamics) remained unclear (Booth et al., 2012; Dagan et al., 2020; Menary et al., 2020). We note that the net North Atlantic surface energy fluxes and NASST well correlate (Figure 1a). This close relationship suggests that the detrended NASST variability is associated with aerosol‐induced changes in surface radiation. Several studies demonstrated that aerosol‐induced changes in ocean circulation (e.g., AMOC) modulates NASST, especially in the North Atlantic warming hole (NAWH) region, where the SST changes are more sensitive to ocean circulation than radiative fluxes changes (Dagan et al., 2020; Menary et al., 2020). Therefore, we split the North Atlantic Ocean region into two subregions, NAWH and its surrounding area (see the green and magenta boxes in Figure 3d). Figure 3e shows that NAWH exhibits a cooling trend, opposite to the warming trend shown in the rest of the North Atlantic Ocean (Figure 3f). Changes in ocean dynamics may lead to the opposite trends of NASST between NAWH and the rest of the North Atlantic Ocean. However, the detrended SST variability is similar between NAWH and the rest of the North Atlantic Ocean. Therefore, we suggest that while SST is sensitive to changes in ocean dynamics in the NAWH region, this is manifested in determining the trend, and the detrended variability is still modulated by changes in surface radiative fluxes. This is consistent with observational data sets (Figures 3e and 3f), although there is a discrepancy in magnitude and a lag between observed and CESM1‐LE NAWH SST.

Our results link the process chain from changing sulfate emissions from Europe and North America, to changes in North Atlantic surface net radiative fluxes, via NASST variability to a shift of ITCZ and changes in West African monsoon, and finally Sahel rainfall variability, as illustrated in Figure 3g.

**Figure 4:**
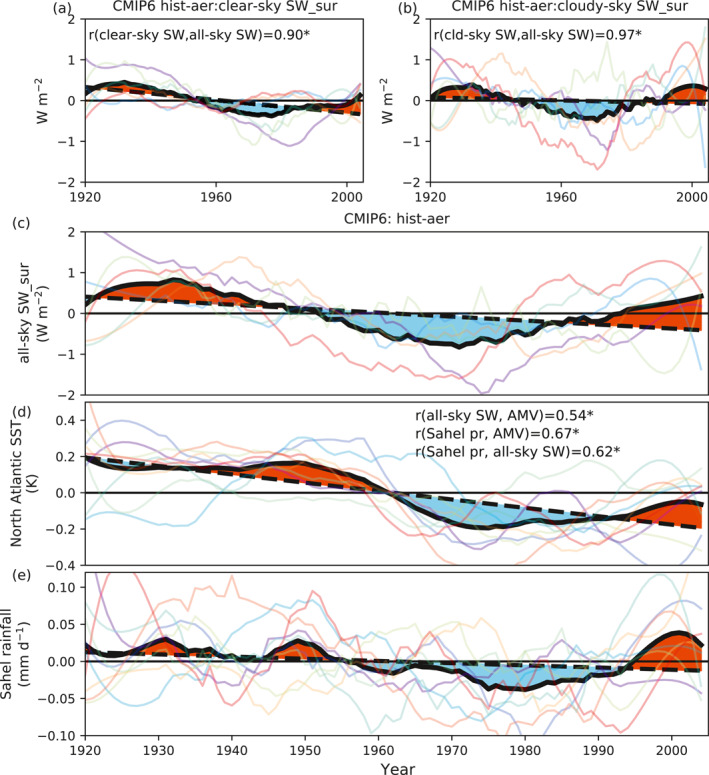
(a–c) Coupled Model Intercomparision Project Phase 6 (CMIP6) hist‐aer experiments derived (a) clear‐sky shortwave surface radiative flux, (b) cloudy‐sky (difference between all‐sky and clear‐sky) shortwave surface radiative flux and (c) all‐sky shortwave surface radiative flux, over the North Atlantic Ocean surface. All radiative fluxes are defined as downward positive. Each colored line indicates a CMIP6 participating model. (d) North Atlantic sea surface temperature (NASST) for hist‐aer experiments. (e) Sahel rainfall anomalies for historical simulations with only anthropogenic aerosol forcing (hist‐aer). All anomalies are relative to the 1920–1925 average. The mark “*” after the correlation coefficient indicates the correlation is significant (*p* value < 0.01). Dashed lines indicate the linear trend and red/blue patches indicate the positive/negative phase.

To strengthen the robustness of our conclusions in the light of model uncertainties, we further analyze CMIP6 historical simulations. We note that while an ensemble mean from eight CMIP6 models cannot entirely eliminate the natural variability, it can significantly reduce its impact. While the individual models suggest various patterns of 20th‐century AMV and Sahel rainfall, the correlation between AMV and Sahel rainfall is high (Figure S6 in Supporting Information S1). Figure 2 has already illustrated an agreement of the CMIP6 historical ensemble mean with the multidecadal detrended variability of 20th‐century NASST and Sahel rainfall found in CEMS‐LE and observations. Furthermore, historical simulations with only anthropogenic aerosol emissions (hist‐aer) also show significant positive correlations among surface radiative fluxes, NASST and Sahel rainfall variability (Figures 4a–4e). In contrast, simulations with only GHGs emissions (hist‐ghg) do not reproduce this pattern (Figure S7 in Supporting Information S1), indicating that anthropogenic aerosol changes cause this multidecadal variability.

Considering that aerosols can reduce surface solar radiation through direct effects and through their interaction with clouds (Boucher et al., 2013), it would be of interest to know which processes (aerosol‐cloud interactions or aerosol‐radiation interactions) contribute more to the variability of NASST and ultimately Sahel rainfall. We note that there might also be cloud feedbacks due to the change of NASST in coupled experiments. CESM1‐LE ensemble‐mean results imply that the multidecadal aerosol effect on NASST is dominated by aerosol‐cloud interactions (around two thirds) rather than aerosol‐radiation interactions (around one third) (Figure S8 in Supporting Information S1). Considering aerosol effects exhibit significant uncertainties among GCMs (Bellouin et al., 2020; Ghan et al., 2016; Zhang et al., 2016), and that CESM‐CAM5 has a larger sensitivity of cloud liquid water path to aerosols (Wang et al., 2012), we also analyze this split across CMIP6 hist‐aer simulations. Clear‐sky and cloudy‐sky surface fluxes shown in Figures 4a and 4b imply that both aerosol direct and indirect effects contribute to this positive‐negative‐positive pattern of detrended net surface shortwave radiative flux (Figure 4c), with similar magnitude. However, we find that the uncertainties (as indicated by model diversity) arise primarily from aerosol‐cloud interactions (Figure 4b), and these uncertainties may also propagate to simulated NASST variability (Figure 4d) and subsequently Sahel rainfall variability (Figure 4e and Figure S9 in Supporting Information S1).

## 4 Conclusions

Previous studies (Biasutti & Giannini, 2006; Dong & Sutton, 2015; Held et al., 2005; Herman et al., 2020; Hirasawa et al., 2020; Palmer, 1986) proposed several drivers to understand the severe drought and the subsequent recovery of Sahel rainfall during the past century. However, the attribution remained ambiguous and model dependent. We assess the temporal evolution of the 20th‐century North Atlantic SST and Sahel rainfall and decompose them into a linear trend and detrended variability. Although the linear trend will be affected by a mix of external forcings and internal variability and the aerosol effect does not stand out alone, we show that anthropogenic aerosol effects modulate the detrended multidecadal variability. Our results robustly demonstrate a chain of processes from aerosol (precursor) emissions, their direct and indirect effects on shortwave radiative fluxes, via North Atlantic SST variability, to changes in the ITCZ position due to the inter‐hemispheric temperature difference, subsequently modulating the West African monsoon and, finally, Sahel rainfall (Figure 3g). This conclusion is consistently supported by independent data from the CESM1‐LE, CanESM2‐LE simulations, CMIP6 models, and observational data sets.

This work suggests that the multidecadal variability of Sahel rainfall is modulated by anthropogenic aerosols. We also explicitly note that other mechanisms, for example, internal variabilities, will contribute, to avoid any ambiguity. The forced variability of North Atlantic SST is contributed by aerosol‐induced changes in surface radiative fluxes rather than changes in ocean circulations. It is also worth noting that aerosol‐cloud interactions contribute most to the inter‐model uncertainties in simulating North Atlantic SST variability and potentially Sahel rainfall (Figure 4). Our results highlight the critical role of anthropogenic aerosols on 20th‐century Sahel rainfall multidecadal variability through their impacts on North Atlantic SST. Therefore, it is essential to correctly simulate regional aerosol radiative effects (especially aerosol‐cloud interactions) for future projections of Sahel rainfall.

## Conflict of Interest

The authors declare no conflicts of interest relevant to this study.

## Supporting information

Supporting Information S1

## Data Availability

The Community Earth System Model 1 large ensemble simulations (CESM1‐LE) data sets are available at https://www.cesm.ucar.edu/projects/community-projects/LENS/. The Canadian Earth System Model 2 large ensemble simulations (CanESM2‐LE) data sets are available at http://crd-data-donnees-rdc.ec.gc.ca/CCCMA/products/CanSISE/output/CCCma/CanESM2/. The CMIP6 models used in this study have been listed in Table S1 in Supporting Information S1. Data sets from the Coupled Model Intercomparision Project Phase 6 are available at https://pcmdi.llnl.gov/CMIP6/. The emission data set of anthropogenic sulfur dioxide (labeled as input4MIPs.CMIP6.CMIP.PNNL‐JGCRI.CEDS‐2017‐05‐18.atmos.mon.SO2‐em‐anthro.gn) is from the input4MIPS project (http://esgf-node.llnl.gov/search/input4mips/?mip_era=CMIP6&activity_id=input4MIPs&institution_id=PNNL-JGCRI&target_mip=CMIP&source_id=CEDS-2017-05-18). Observations are available at the following websites: HadISST (https://www.metoffice.gov.uk/hadobs/hadisst/) and NOAA SST (https://psl.noaa.gov/data/gridded/data.noaa.ersst.v5.html), CRU (https://crudata.uea.ac.uk/cru/data/hrg/cru_ts_4.03/).
