# Supplementary material for: Anthropogenic Aerosols Modulated 20th‐Century Sahel Rainfall Variability Via Their Impacts on North Atlantic Sea Surface Temperature
Source: Geophys Res Lett. 2021 Dec 28;49(1):e2021GL095629. doi: 10.1029/2021GL095629 (PMC9287015; doi:10.1029/2021GL095629)
Supplement: Supplementary file 1 — Supporting Information S1 [file GRL-49-0-s001.docx]

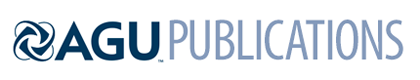


*Geophysical Research Letters*

Supporting Information for

**Anthropogenic aerosols modulated twentieth-century Sahel rainfall variability via their impacts on North Atlantic sea surface temperature**

Shipeng Zhang1*, Philip Stier1, Guy Dagan2, and Minghuai Wang3

1 Atmospheric, Oceanic and Planetary Physics, Department of Physics, University of Oxford, UK.

2 Institute of Earth Sciences, Hebrew University of Jerusalem, Israel.

3 Joint International Research Laboratory of Atmospheric and Earth System Sciences and School of Atmospheric Sciences, Nanjing University, China.

*Correspondence to: Shipeng Zhang (shipeng.zhang@physcis.ox.ac.uk)

**Contents of this file**

Figures S1 to S9

Tables S1

Supporting information contains the CESM-LE simulated Sahel rainfall and NASST for all-forcing and XGHG experiments (Figure S1), the normalised anthropogenic sulphur dioxide emission from different regions (Figure S2), the geographical pattern of anthropogenic sulphur dioxide emission at year 1900, 1950, 1975 and 2000 (Figure S3), the similar results shown by CanESM2-LE (Figure S4), the geographical pattern of NASST and Sahel rainfall for positive AMV phase period in CESM-LE all forcing experiments and observation (Figure S5), the simulated historical NASST and Sahel rainfall in CMIP6 models (Figure S6), and the CMIP6 ensemble-mean Sahel rainfall for hist-ghg and hist-nat experiments (Figure S7), the decomposition of cloudy-sky and clear-sky contributions to CESM-LE downward shortwave radiative flux over the North Atlantic sea surface (Figure S8), and the scatter plot of each component involved in the process chain, simulated in each CMIP6 model (Figure S9).

Supporting information also includes the information of participating CMIP6 models used in this study (Table S1).


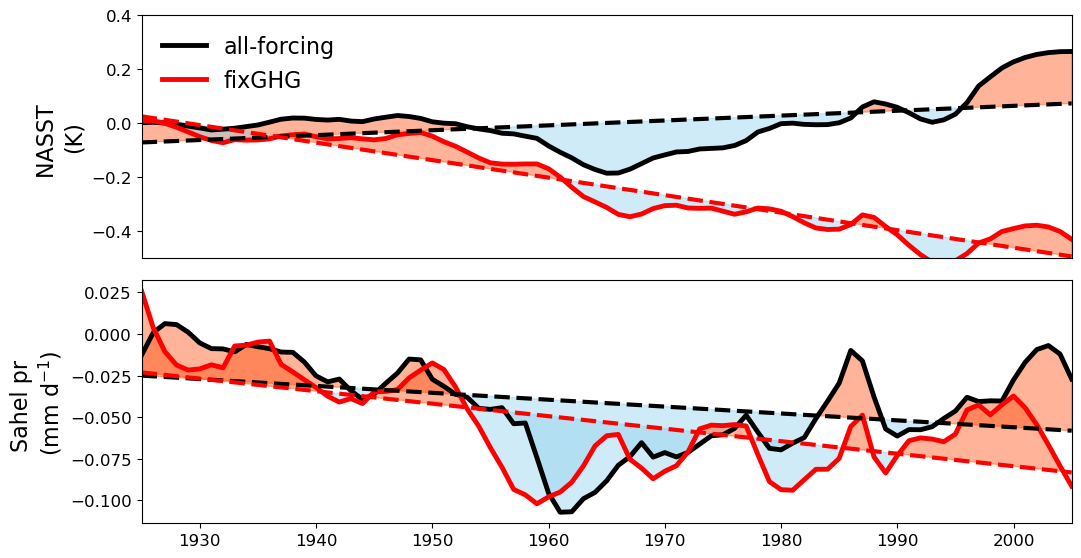


Figure S1. CESM-LE simulated (**a**) North Atlantic (7.5°-75°W, 10°-60°N) SST and (**b**) Sahel rainfall for All-forcing simulations (black line) and fixGHG simulations (red line). All datasets are shown as the anomalies (10 years low-pass filtered) relative to the 1920-1925 average. Dashed lines indicate the linear trend and red/blue patches indicate the positive/negative phase of detrended datasets. Thin lines indicate each ensemble members.

*
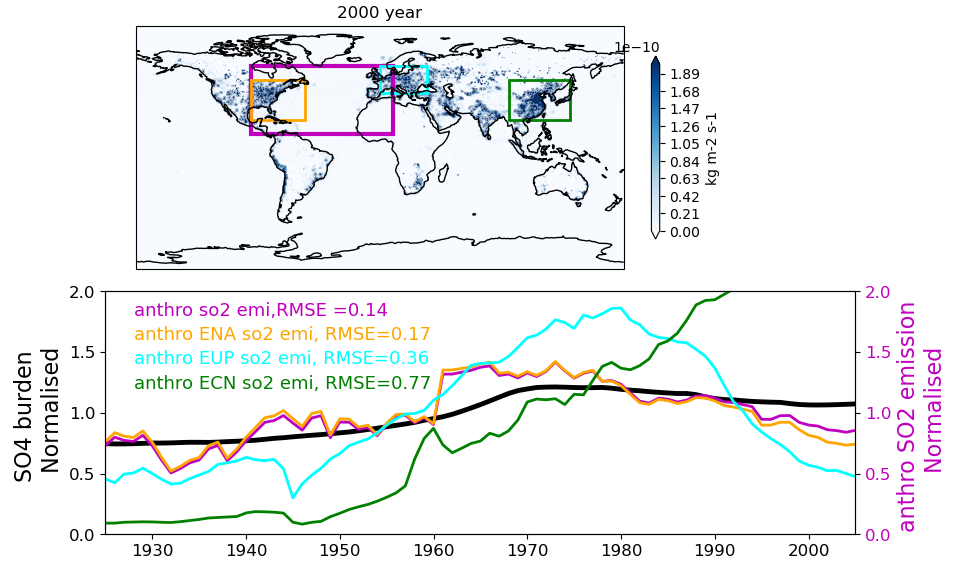
*

Figure S2. (upper panel) Normalised (divided by averages) anthropogenic sulphur dioxide emissions provided by input4MIPS (Hoesly et al., 2018) at year 2000. (lower panel) Normalised (divided by averages) CESM-LE all-forcing experiment simulated sulphate aerosol burden over the North Atlantic Ocean (black line) and 20th-century anthropogenic sulphur dioxide emissions from the region chosen in Figure 1b (purple line), North-East America (orange line), Europe (cyan line), and East China (green line), respectively. The numbers indicate the root-mean-square deviation (RMSE) between regional anthropogenic SO2 emissions and SO4 burden over the North Atlantic Ocean.


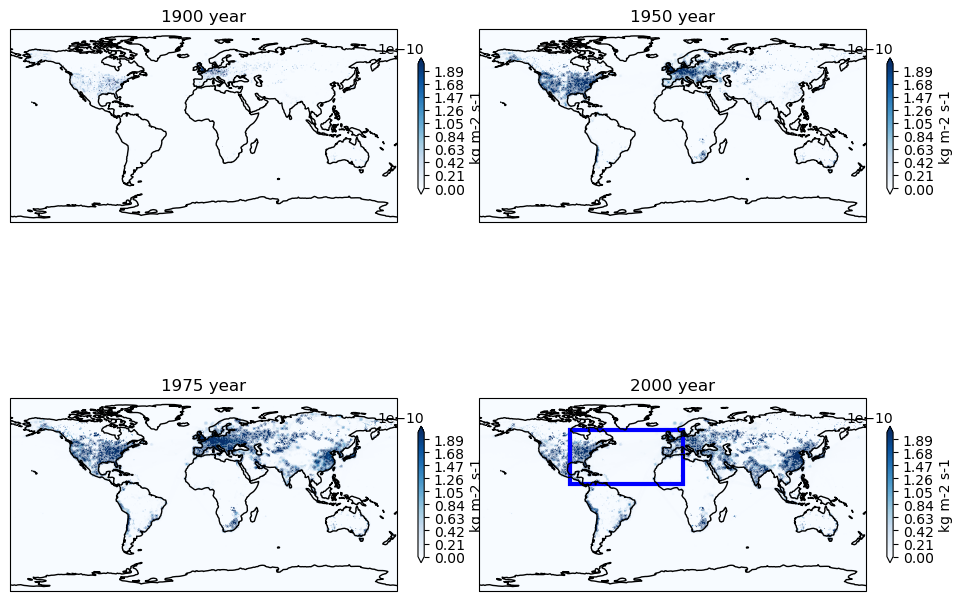


Figure S3. The anthropogenic sulphur dioxide emission provided by input4MIPS at year 1900, 1950, 1975 and 2000 respectively. The blue box indicates the region where the emission averaged over in Figure 1b.

**
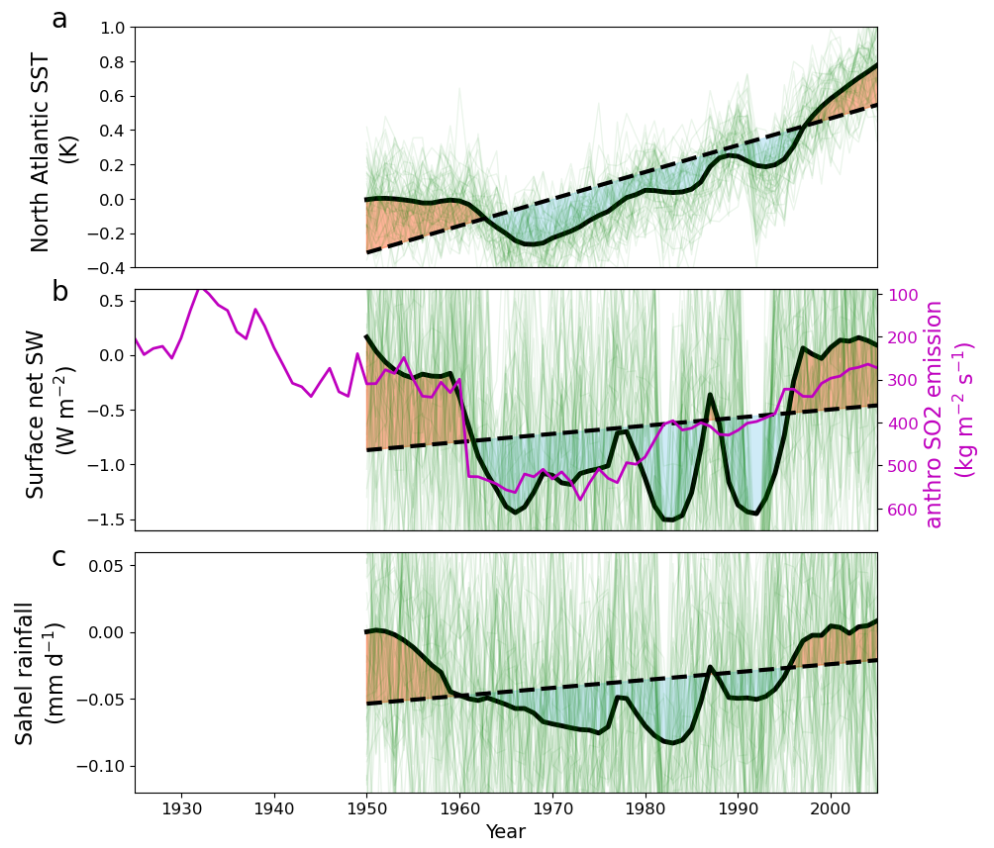
**

Figure S4. Similar to Figure 1 but with CanESM2-LE data. (a) CanESM2-LE simulated North Atlantic (7.5°-75°W, 10°-60°N) SST for all-forcing simulations (black line). (b) CanESM2-LE all-forcing experiments simulated net surface solar radiative flux (black line). The purple line indicates the 20th-century anthropogenic sulphur dioxide emissions from the North Atlantic Ocean as well as East North America and West Europe (notice the y-axis has been reversed). (c) CanESM2-LE simulated ensemble-mean Sahel rainfall for all-forcing experiments. All datasets are shown as anomalies (10 years low-pass filtered) relative to the 1950-1955 average. Dashed lines indicate the linear trend, and red/blue patches indicate the positive/negative phase of detrended datasets. Thin green lines are the individual members from CanESM2-LE.


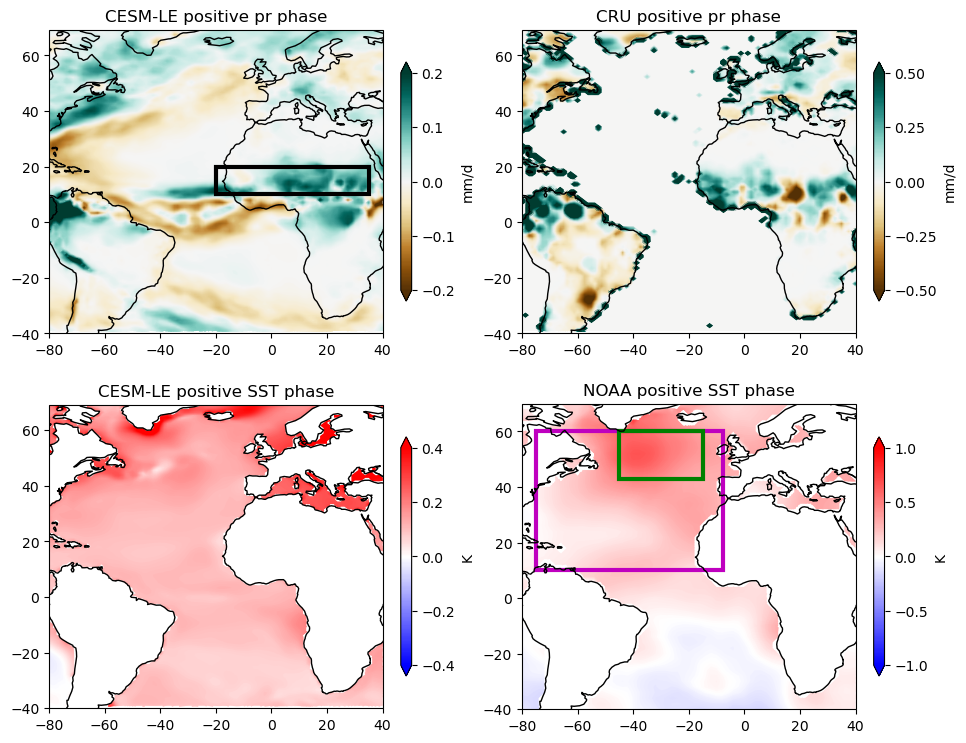


Figure S5. Same as Figure3 but for the positive phase (year 1995 to 2005) of detrend NASST, and the pattern of precipitation at the same period.


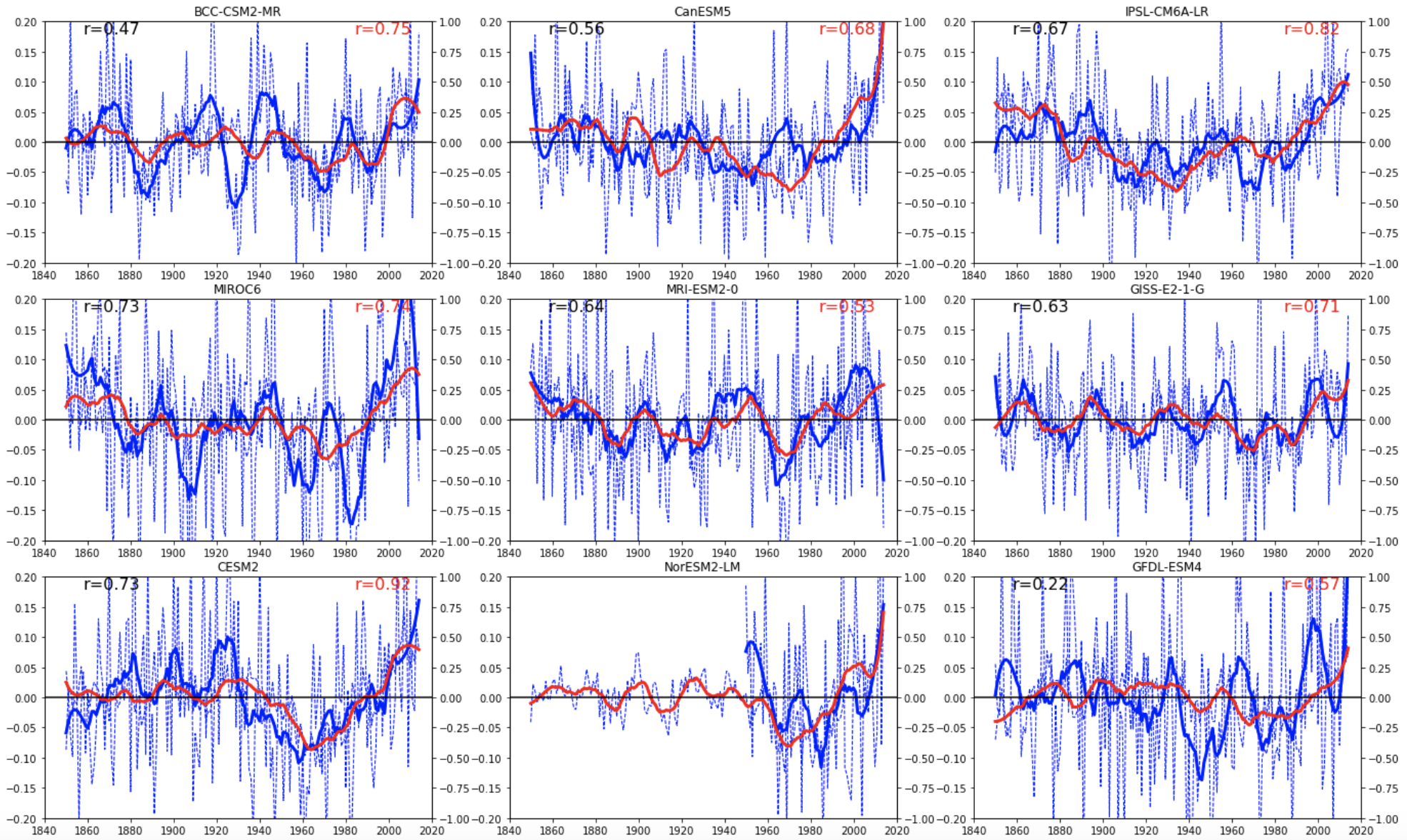


Figure S6. The relationship between North Atlantic sea surface temperature (anomaly, red line, right y-axis, unit k) and Sahel rainfall (anomaly, blue line, left y-axis, unit mm/d) in each CMIP6 model. Black numbers on the right indicate the correlation coefficients between North Atlantic sea surface temperature and Sahel rainfall since 1850, while red numbers indicate the correlation coefficients since 1920.


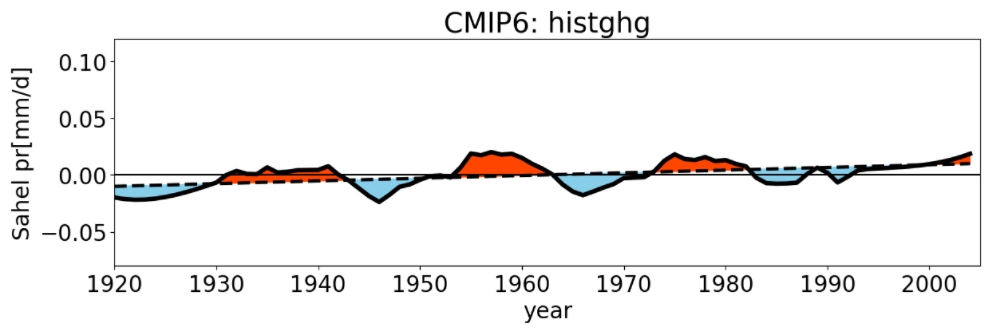

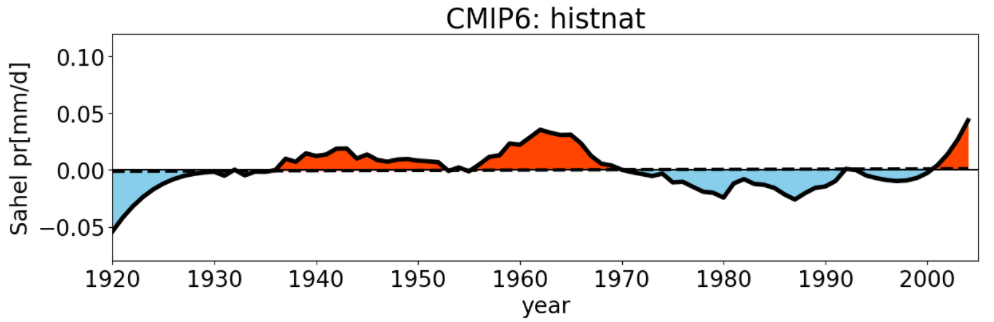


Figure S7. CMIP6 ensemble-mean Sahel rainfall for (a) hist-ghg and (b) hist-nat experiments.


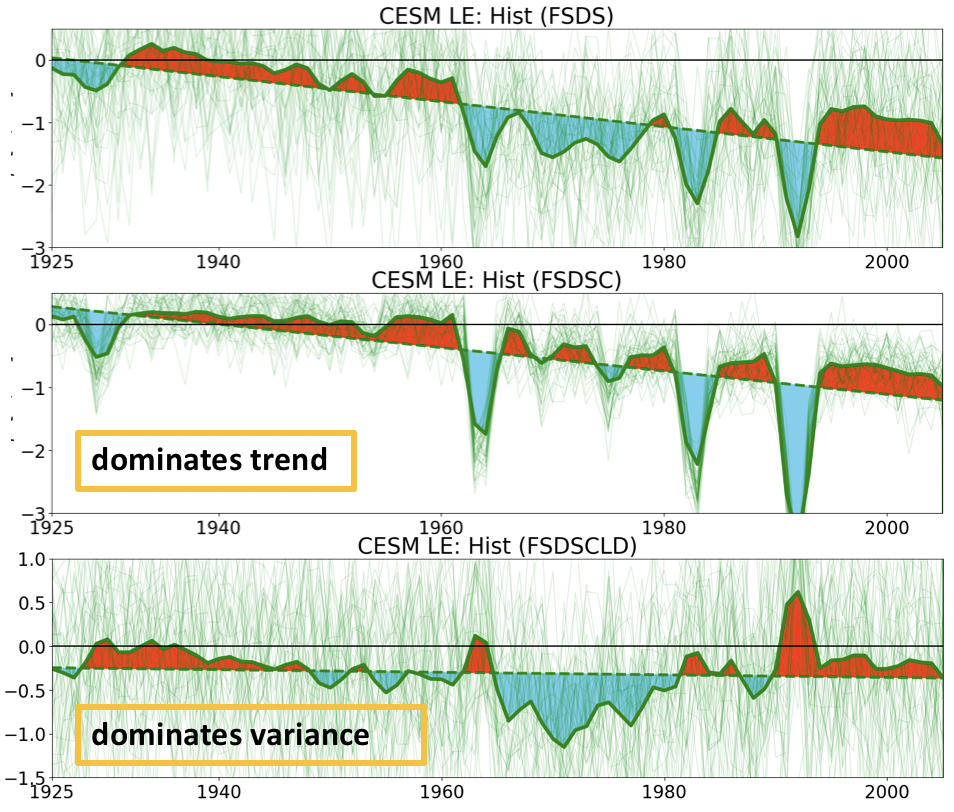


Figure S8. (**a**) all-sky downward shortwave radiative flux, (**b**) clear-sky downward shortwave radiative flux and (**c**) cloudy-sky downward shortwave radiative flux over the North Atlantic Ocean surface. All radiative fluxes are defined as downward positive. Thin lines indicate each ensemble members.


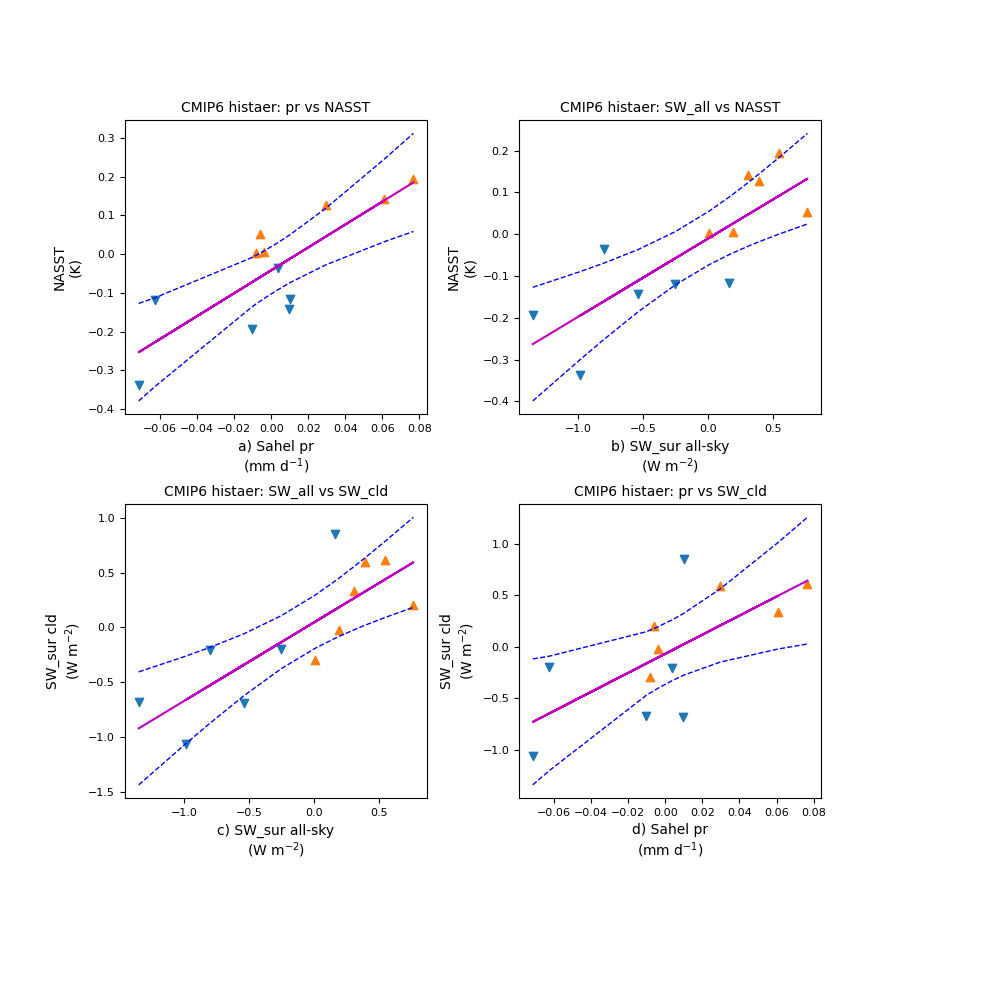


Figure S9. The scatter plot of a) Sahel rainfall and NASST; (b) all-sky surface net shortwave flux and NASST; (c) all-sky surface net shortwave flux and cloudy-sky surface net shortwave flux; (d) Sahel rainfall and cloudy-sky surface net shortwave flux. Each triangle indicates individual CMIP6 model results sampled over positive phase (orange triangles) and negative phase (blue triangles).

Table S1. The information of participating CMIP6 models used in this study

| Model name | Institution | Resolution | path |
| --- | --- | --- | --- |
| BCC-CSM2-MR | Beijing Climate Center, China | 320x160 | gs://cmip6/DAMIP/BCC/BCC-CSM2-MR/ |
| CanESM5 | Canadian Centre fro Climate Modelling and Analysis (CCCma), Canada | 128x64 | gs://cmip6/DAMIP/CCCma/CanESM5/ |
| IPSL-CM6A-LR | L'Institut Pierre-Simon Laplace (IPSL), France | 144x143 | gs://cmip6/CMIP6/DAMIP/IPSL/IPSL-CM6A-LR/ |
| MIROC6 | Model for Interdisciplinary Research on Climate (MIROC), Japan | 256x128 | gs://cmip6/CMIP6/DAMIP/MIROC/MIROC6/ |
| MRI-ESM2-0 | Meteorological Research Institute (MRI), Japan | 320x160 | gs://cmip6/CMIP6/DAMIP/MRI/MRI-ESM2-0/ |
| GISS-E2-1-G | NASA Goddard Institute for Space Studies  (GISS), United States | 144x90 | gs://cmip6/CMIP6/DAMIP/NASA-GISS/GISS-E2-1-G |
| CESM2 | National Center for Atmospheric Research (NCAR), United States | 288x192 | gs://cmip6/CMIP6/DAMIP/NCAR/CESM2/ |
| NorESM2-LM | Norwegian Climate Centre (NCC), Norway | 144x96 | gs://cmip6/CMIP6/DAMIP/NCC/NorESM2-LM/ |
| GFDL-ESM4 | Geophysical Fluid Dynamics Laboratory (GFDL), United States | 288x180 | gs://cmip6/CMIP6/DAMIP/NOAA-GFDL/GFDL-ESM4/ |
